# Sinapic Acid and Sinapate Esters in *Brassica*: Innate Accumulation, Biosynthesis, Accessibility *via* Chemical Synthesis or Recovery From Biomass, and Biological Activities

**DOI:** 10.3389/fchem.2021.664602

**Published:** 2021-05-14

**Authors:** V. P. Thinh Nguyen, Jon D. Stewart, Irina Ioannou, Florent Allais

**Affiliations:** ^1^ URD Agro-Biotechnologies Industrielles (ABI), CEBB, AgroParisTech, Pomacle, France; ^2^ Department of Chemistry, University of Florida, Gainesville, FL, United States

**Keywords:** Brassica, *p*-hydroxycinnamic acids, sinapic acid, sinapine, sinapoyl glucose, sinapoyl malate

## Abstract

Sinapic acid (SinA) and corresponding esters are secondary metabolites abundantly found in plants of Brassica family. Belonging to the family of *p*-hydroxycinnamic acids, SinA and its esters analogues are present in different plant parts and involved in multiple biological processes *in planta*. Moreover, these metabolites are also found in relatively large quantities in agro-industrial wastes. Nowadays, these metabolites are increasingly drawing attention due to their bioactivities which include antioxidant, anti-microbial, anti-cancer and UV filtering activities. As a result, these metabolites find applications in pharmaceutical, cosmetic and food industries. In this context, this article reviews innate occurrence, biosynthesis, accessibility *via* chemical synthesis or direct extraction from agro-industrial wastes. Biological activities of SinA and its main corresponding esters will also be discussed.

## Introduction

*p*-Hydroxycinnamic acids represent one of the most widely distributed chemicals in the plant kingdom, along with other phenylpropanoids such as flavonoids, stilbenes, and lignans. *p*-Hydroxycinnamic acids occur in fruits, vegetables, cereals, and beverages and are involved in plant tissue development and response to external stress (Nicholson and Hammerschmidt, 1992; Beckman, 2000).

Primary roles of *p*-hydroxycinnamic acids in different parts of plants include coloration of flowers that attract pollinating animals, protection from injurious UV radiation, natural aromas and tastes that defend against predators, resistances to pathogens, and enhancing the host plants by affecting the growth of other, neighboring plants (Beckman, 2000).

**FIGURE 1:**
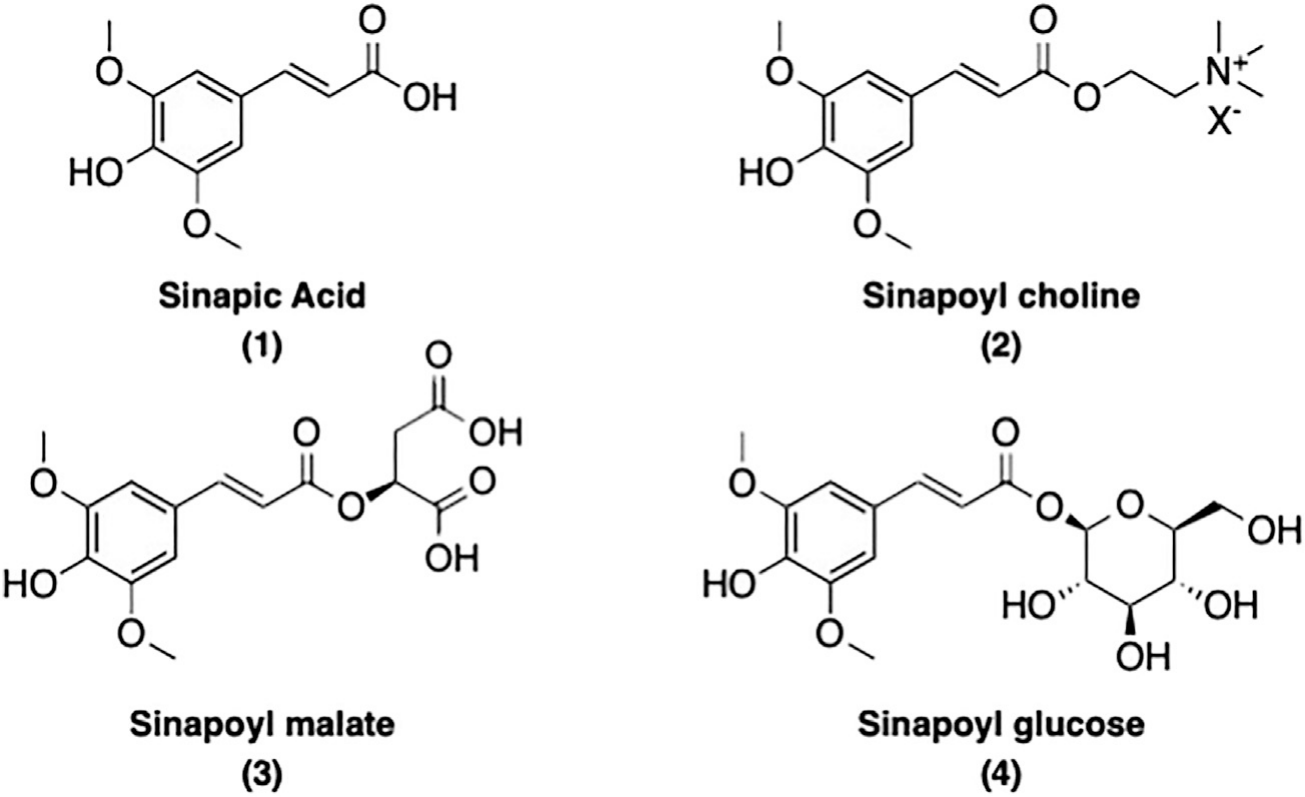
Structures of SinA and its main corresponding esters present in this review.

Sinapoyl esters (SinEs) are the most important *p*-hydroxycinnamoyl esters present in plants of the *Brassicaceae* species (Nićiforović and Abramovič, 2014; Chen, 2016). SinEs possess a *p*-hydroxyphenol moiety with two methoxy substituents at the *meta*-positions. Along with its free acid form sinapic acid (SinA) (**1**), many SinEs have been found in plants, such as sinapoyl choline [also known as sinapine (**2**)], sinapoyl malate (**3**), sinapoyl glucose (**4**), and many more. The structure of SinA and its major corresponding esters present in this review are illustrated and listed in Figure 1.

SinEs have various biological properties such as antimicrobial (Maddox et al., 2010; Engels et al., 2012), anti-inflammatory (Yun et al., 2008; Oueslati et al., 2012), anticancer (Oueslati et al., 2012; Raj Preeth et al., 2019; Boeing et al., 2020), and anti-anxiety activities (Yoon et al., 2007). Moreover, it is suggested that SinEs could be used as food and cosmetic additives, as well as bioactive compounds in the pharmaceutical industry (Nićiforović and Abramovič, 2014). SinA, the carboxylic acid form of SinE, could be employed as a building block for the design of renewable monomers and polymers (Jaufurally et al., 2016; Janvier et al., 2017; Hollande et al., 2019).

Here, we review the innate accumulation of *p*-hydroxycinnamic acid and its derivatives in *Brassica* plants, mainly focusing on SinA and SinE. Moreover, we will discuss their biosynthesis, accessibility *via* chemical synthesis or direct extraction, and biological activities.

## Innate Accumulation of Sinapic Acid and Its Derivatives in Brassica

Phenylpropanoids are omnipresent in the plant kingdom and have been identified in a wide variety of edible plants including fruits, vegetables, cereals, and spices. The concentration of SinA and its derivatives varies from one species to another. For instance, strawberries, *Fragaria ananassa L.*, was reported to possess the highest concentration of SinE (up to 450.30 μg/g of biomass), while the lowest concentration was determined in rye *Secale cereale L.* (a few µg/g of biomass) (Nićiforović and Abramovič, 2014). The review by Niciforovic and Abramovic provides a detailed report on the natural occurrence of these phenolic compounds (Nićiforović and Abramovič, 2014). Within the *Brassicaceae* vegetables, SinA and its derivatives are ubiquitously present in both free and esterified forms. Many SinEs have been identified in different species from *Brassica* family (Lin and Harnly, 2010; Cartea et al., 2011). Generally, the concentration of naturally occurring SinA appears to be lower than its choline ester, sinapine. The concentrations of SinEs, mainly sinapine, range from 8 to 10.4 mg/g of biomass, whereas SinA ranges from 0.49 to 2.49 mg/g of biomass (Thiel et al., 2015; Odinot et al., 2017; Reungoat et al., 2020).

The accumulation of phenolic compounds has been probed within different parts of *Brassica* plants. Many SinEs have been identified in edible parts such as leaves, stems, flower buds, and roots (Fernandes et al., 2007). Malate derivatives were determined to be the main *p*-hydroxycinnamic esters presented in the leaves of pak choi *Brassica campestris* L subsp. *chinensis* and Chinese mustard *Brassica juncea* Coss (Harbaum et al., 2008). SinA and its esters have also been found in large amounts within rapeseed seeds and in their defatted residues. In their study of rapeseed meal, Laguna et al. reported that the SinA concentration in non-industrial and industrial meals, after alkaline hydrolysis, was up to 14.0 and 10.5 mg/g of dry matter, respectively (Laguna et al., 2019). Another study on aqueous ethanol extracts from mustard bran (*B. juncea*) reported that the sinapine concentration was up to 8.7 mg/g of dry matter (Reungoat et al., 2020).

The accumulation of SinA and its derivatives, along with other phenylpropanoids, is believed to favor the adaptation process in plants under environmental stresses. By soaking *B. juncea* seeds prior to germination in 24-epibrassinolide, Sharma et al. observed enhanced accumulation of phenolic compounds in the presence of imidacloprid (Sharma et al., 2016). The levels of SinA and its derivatives in seedlings that grew from soaked seeds were increased by over 100% compared to seedlings derived from untreated seeds. The accumulation of SinA and its derivatives also occurred under biotic stress including insect attack and pathogen infection. Gunnaiah et al. observed that the up-regulation of phenylpropanoid biosynthesis occurred in wheat infested with *Fusarium graminearum*, a fungal plant pathogen (Gunnaiah et al., 2012). An increased cell wall thickness prompted an excessive accumulation of SinA and its derivatives in infested plants, which is thought to be a physiological response to biotic stress. It was furthermore suggested that *Brassica* plants accumulate phenolics and other metabolites to enhance survival rates against environmental stresses, which is in agreement with Beckman et al.’s suggestion (Beckman, 2000).

The involvement of these secondary metabolites in response to environmental stresses, however, exacts a cost to the plants with regard to the energy devoted to accumulating phenolic compounds, which leads to lower growth rates. In the study conducted by Moreno et al., chinese cabbage (*Brassica rapa L.* subsp. *Pekinenesis*) grown under sub-optimal conditions accumulated higher phenolic concentrations than those grown under optimal conditions (Moreno et al., 2003). The weights of plants grown under sub-optimal conditions were, as a result, lower than those grown under optimal conditions.

The accumulation of SinA and its derivatives varies with the growth environment since these modulate the physiological state of the plants. We therefore suggest that adverse environmental factors should be included in future studies in order to anticipate potential over-accumulation of these secondary metabolites. The concentration of SinA and its derivatives in plant can also be used as an indicator to monitor plant growth and the effects of growth conditions on plant development.

## Biosynthesis of Sinapic Acid and Derivatives

Plant growth depends on environmental conditions and accumulating phenolics enables plants to survive under sub-optimal growth conditions (Blokhina et al., 2003; Toscano et al., 2019). SinA and its derivatives, along with other secondary metabolites, are biosynthesized by plants *via* a set of chemical reactions (Vogt, 2010). Studying these pathways within plants will therefore allow us to understand how environmental stresses affect phenolic accumulation generally, and more specifically, SinA and SinE in *Brassica* plants. In recent years, the biosynthesis of these secondary metabolites has been extensively studied thanks to the advanced development of model plants including *Arabidopsis thaliana*, a member of the *Brassica* family (Vogt, 2010; Fraser and Chapple, 2011).

**FIGURE 2:**
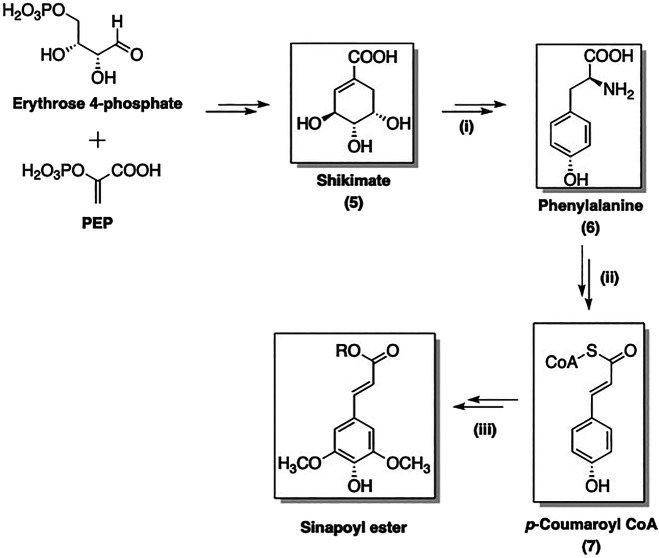
Three main stages of SinE biosynthesis: (i): Formation of Phe; (ii): Formation 4-coumaroyl CoA intermediate; (iii) Formation of sinapoyl esters.

The biosynthesis of SinA derivatives involves the phenylpropanoid pathway *via* the formation of shikimate intermediate (**5**). This route is composed of three sequential stages: (i) formation of phenylalanine (**6**) *via* the shikimate pathway; (ii) non-oxidative deamination of **6** followed by oxygenation to yield activated *p*-coumaroyl CoA (**7**); (iii) further transformations of **7** to afford a broad range of SinEs (Vogt, 2010). As an example, the biosynthesis of SinE from erythrose 4-phosphate and phosphoenolpyruvate (PEP) is shown in Figure 2.

### Formation of Phenylalanine Following Shikimate Pathway (i)

**FIGURE 3:**
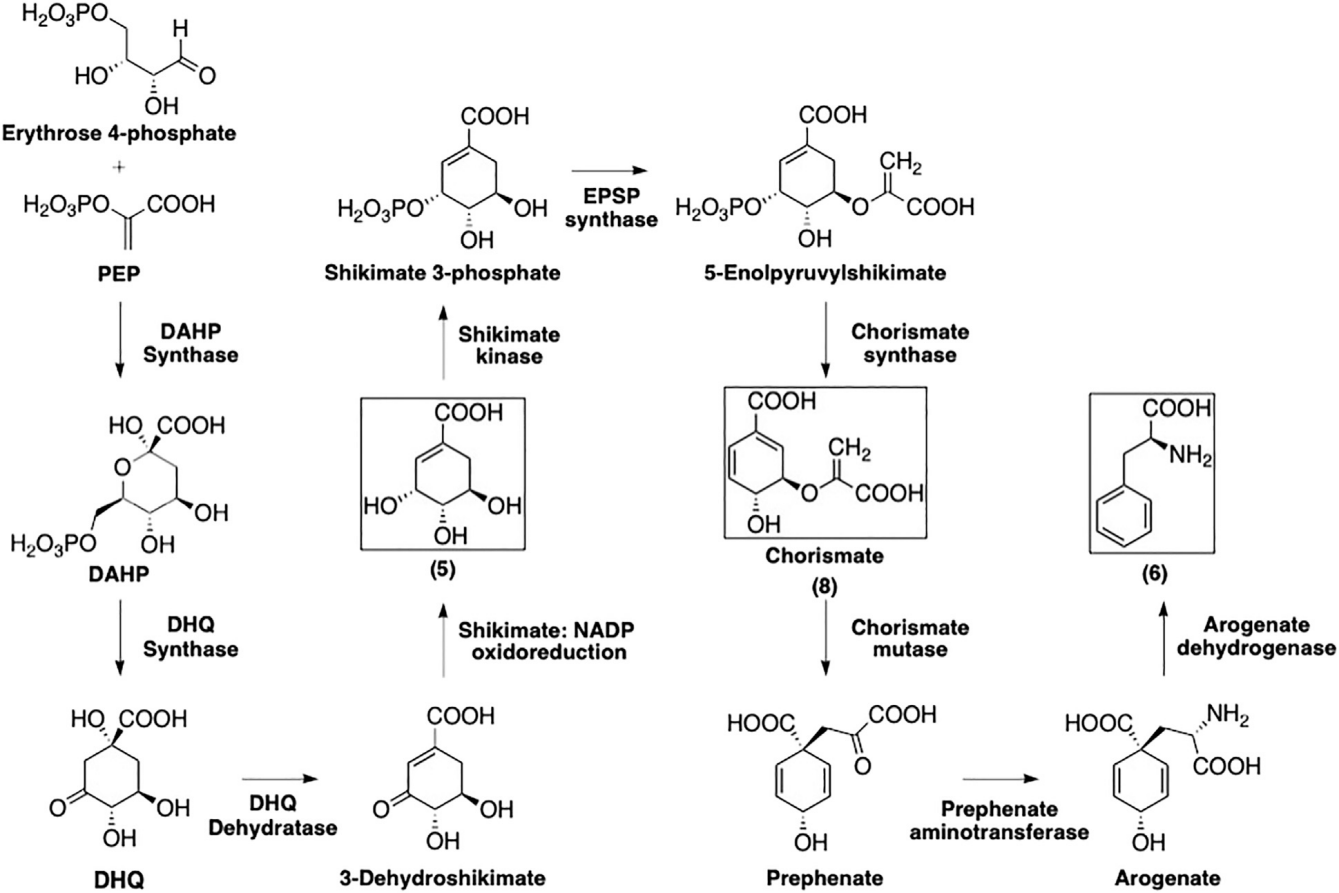
Formation of phenylalanine following the shikimate pathway. Squared boxes indicate relevant intermediates.

As depicted in Figure 3, the formation of phenylalanine (**6**) starts with the 3-deoxy-D-heptulosonate 7-phosphate (DAHP) synthase-catalyzed condensation of PEP and erythrose 4-phosphate to afford DAHP (Schmid and Amrhein, 1995). The latter is then transformed by 3-dehydroquinate synthase into 3-dehydroquinate (DHQ) that is subsequently dehydrogenated and reduced by 3-dehydroquinate dehydratase and the shikimate:nicotinamide adenine dinucleotide phosphate (NADP) oxidoreductase, respectively, to afford shikimate (**5**).

The multi-step conversion of **5** to phenylalanine requires its conversion to chorismate (**8**). Shikimate is phosphorylated by shikimate kinase to yield shikimate 3-phosphate. 5-Enolpyruvylshikimate 3-phosphate (EPSP) synthase then installs a phosphoenolpyruvate group at the 5-position. Finally, chorismate synthase eliminates the phosphate group to afford **8**.

Chorismate (**8**) is converted to prephenate *via* a chorismate mutase-catalyzed Claisen rearrangement of the enolpyruvyl side chain. Prephenate aminotransferase installs the amino group to yield arogenate, then this is simultaneously decarboxylated and dehydrated by arogenate dehydratase to yield **6**.

The formation of aromatic amino acids from PEP and erythrose 4-phosphate has been well studied and many of these enzymes have been isolated and characterized (Herrmann and Weaver, 1999; Maeda and Dudareva, 2012). Detailed discussions of regulation and mechanisms for each of the enzymes involved in the shikimate pathway can be found in a number of previously published reviews (Schmid and Amrhein, 1995; Herrmann and Weaver, 1999; Maeda and Dudareva, 2012).

### Formation of 4-Coumaroyl CoA (ii)

**FIGURE 4:**
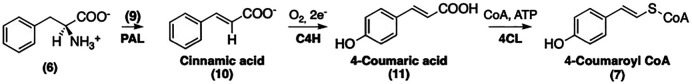
The formation of 4-coumaroyl CoA.

The conversion of phenylalanine (**6**) to 4-coumaroyl CoA (**7**) requires consecutive modifications by phenylalanine ammonia lyase (PAL), cinnamate 4-hydroxylase (C4H), and 4-coumaroyl CoA-Ligase (4CL) (Vogt, 2010). The conversion of 4-coumaroyl CoA to its CoA-linked ester is illustrated in Figure 4.

**FIGURE 5:**
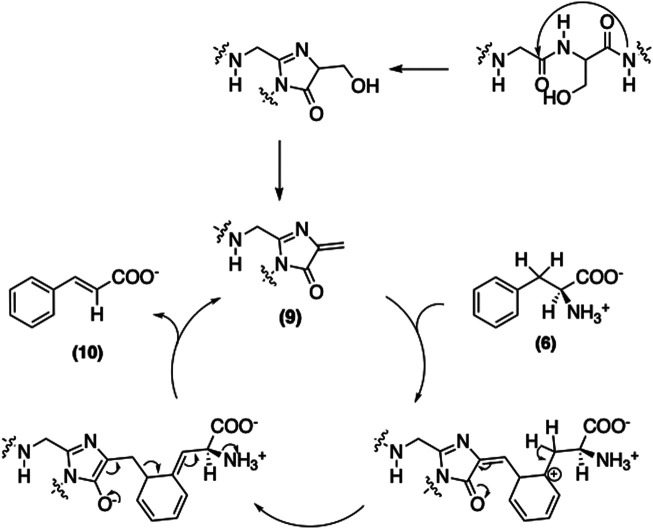
Mechanism of the deamination of phenylalanine catalyzed by PAL.

The first step of this pathway involves the non-oxidative deamination of phenylalanine (**6**) catalyzed by PAL (Koukol and Conn, 1961). The proposed mechanism of PAL is similar to that of histidine ammonia lyase (MacDonald and D’Cunha, 2007). Although no exogenous cofactor is required, an electrophile is still needed for the deamination; hence, the enzyme contains a 3,5-dihydro-5-methyldiene-4H-imidazol-4-one (**9**) moiety, formed by the cyclization and elimination of water from the inner tripeptide Ala-Ser-Gly (Jun et al., 2018). The mechanism of this conversion is shown in Figure 5.

**FIGURE 6:**
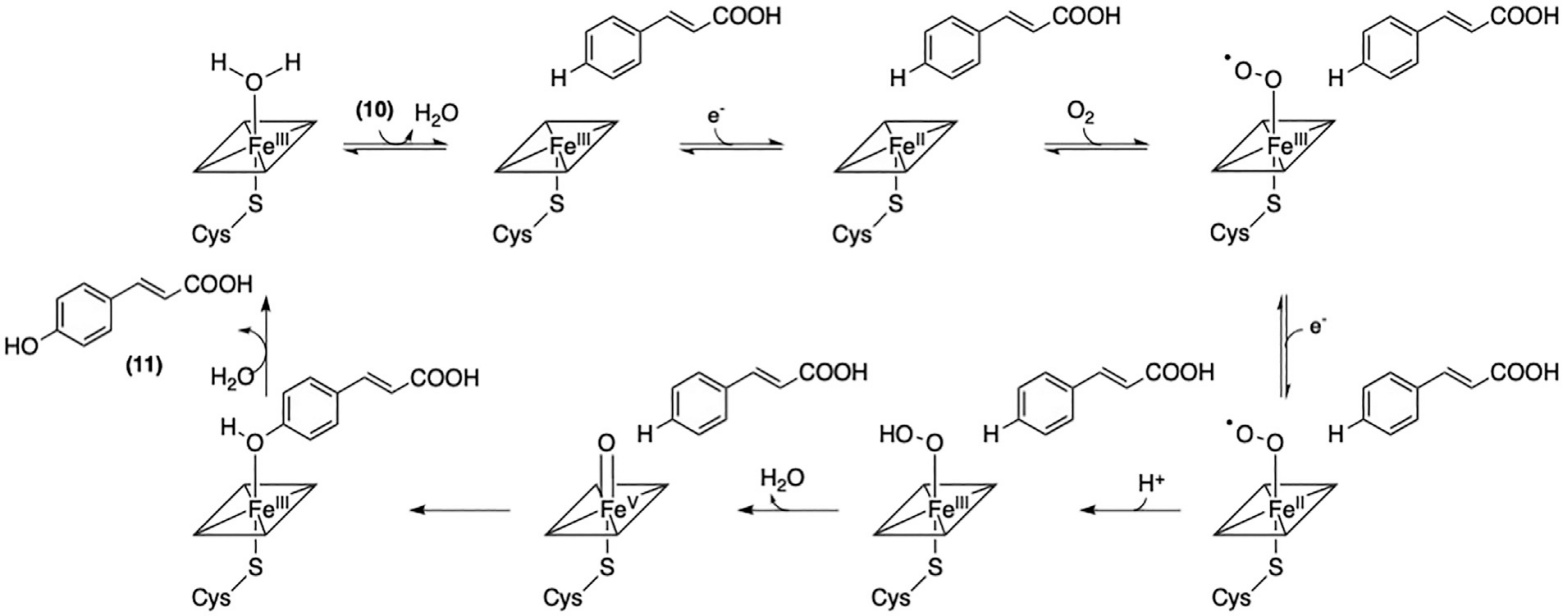
Mechanism of the formation of *p*-coumaric acid catalyzed by C4H.

C4H belongs to the CYP73A family of P450 enzymes and catalyzes the hydroxylation of cinnamic acid (**10**) at the 4-position, yielding 4-coumaric acid (**11**). This transformation requires NADPH-cytochrome P450 reductase, which acts as an electron donor (Werck-Reichhart and Feyereisen, 2000). The crystal structure of C4H from *Sorghum bicolor* (PDB accession number 6VBY) was recently solved and provides critical structural insights into the substrate specificity of this enzyme (Zhang et al., 2020). The mechanism of the C4H-catalyzed transformation of **10** into 4-coumaric acid (**11**) is described in Figure 6.

The final step of this biosynthetic pathway involves the 4CL-mediated conversion of (**11**) into the corresponding Coenzyme-A thioester (**7**). A reaction mechanism has been proposed by Knobloch and Hahlbrock (Knobloch and Hahlbrock, 1975). As depicted in Figure 4, the activation of **11** requires ATP and a CoA unit. The substrate specificity of 4CL has been well studied by Lindermayr et al. (Lindermayr et al., 2003); and. these authors have reported that recombinant 4CL can utilize several different *p*-hydroxycinnamic acids besides **11** including caffeic acid, ferulic acid, and SinA to afford the corresponding CoA-linked thioesters. The recently published crystal structure of 4CL provided further insight into this enzyme with regard to its substrate specificity (Li and Nair, 2015). It is noteworthy that 4CL isoforms also contribute to the biosynthesis of lignin and other secondary metabolites in addition to its involvement in the biosynthesis of SinEs (Goujon et al., 2003; Soubeyrand et al., 2019). The mechanism of the conversion of **10** into **11** is described in Figure 6.

### Formation of Sinapic Acids and Derivatives (iii)

**FIGURE 7:**
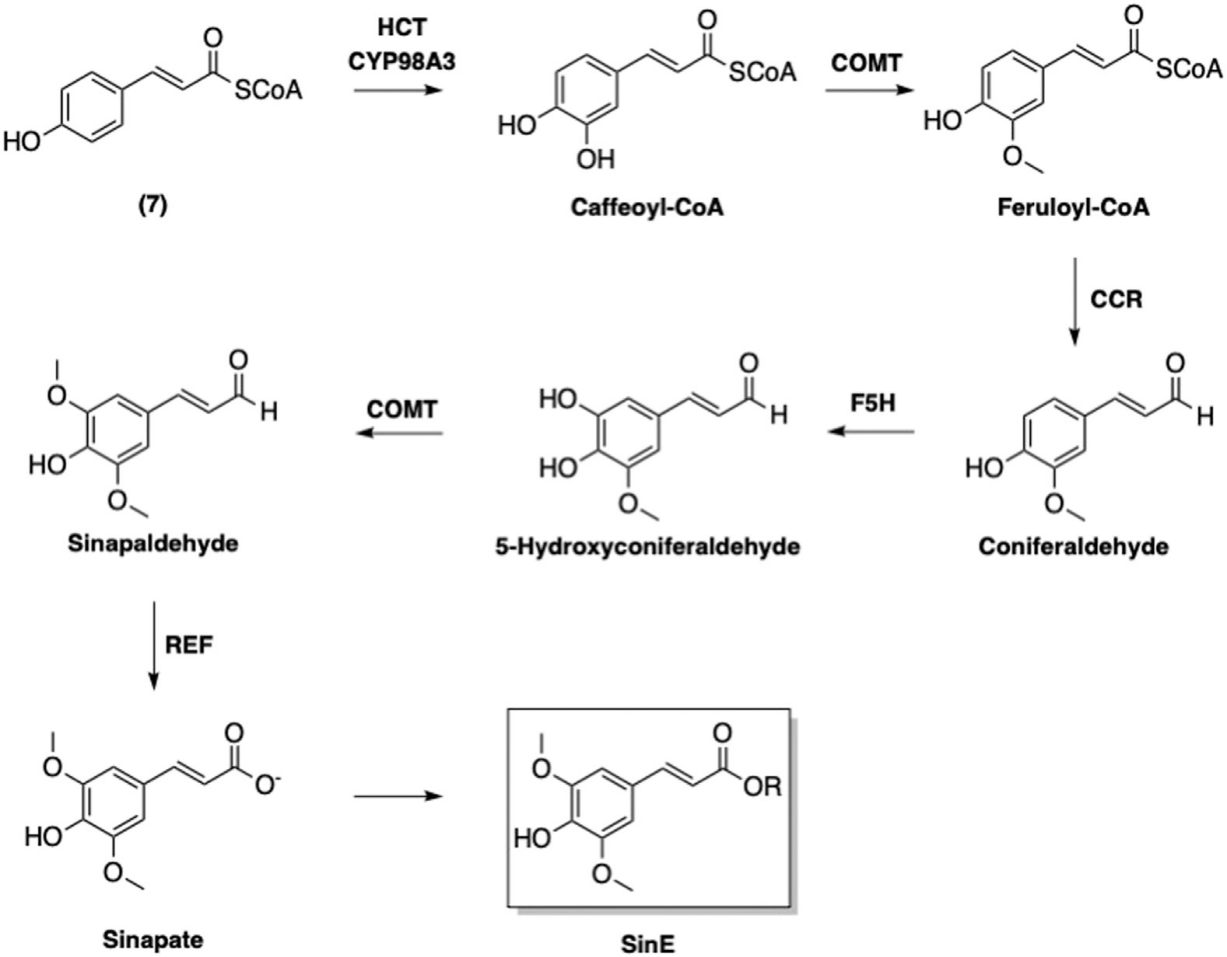
Biosynthesis of sinapoyl esters. HCT: *p*-Hydroxycinnamoyl-CoA: quinate shikimate *p*-hydroxycinnamoyltransferase; COMT: Caffeic acid *O*-methyltransferase; CCR: Cinnamoyl-CoA reductase; F5H: Ferulate 5-hydroxylase; REF: Reduced Epidermal Fluorescence Aldehyde Dehydrogenase.

**FIGURE 8:**
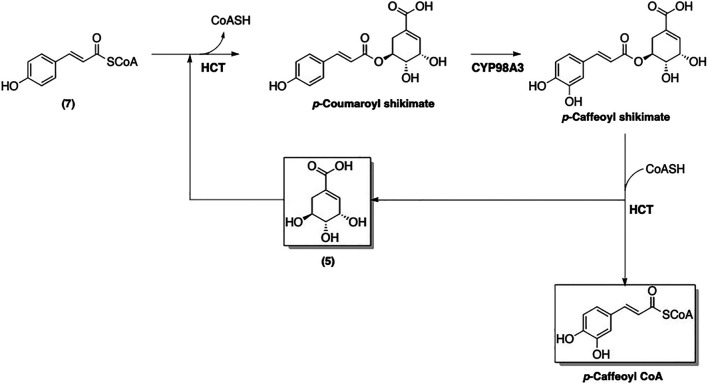
Transformation of 4-coumaroyl CoA (**7**) into caffeoyl CoA.

An enzyme-catalyzed conversions of 4-coumaroyl CoA (**7**) to other phenolic-CoA esters following pathway of phenylpropanoid biosynthesis was suggested (Figure 7) (Boerjan et al., 2003). The first step of this biosynthetic pathway involves adding a hydroxyl group at the 3-position, which converts **7** to caffeoyl-CoA (Figure 8). Interestingly, this modification is catalyzed by *p*-hydroxycinnamoyl-CoA: quinate shikimate *p*-hydroxycinnamoyltransferase (HCT), which also catalyzes a condensation of **7** with **5** to form the corresponding *p*-coumaroyl-shikimate ester (Matsuno et al., 2009). A hydroxyl group is then added by CYP98A3 to afford the caffeoyl-shikimate ester. The transformation of caffeoyl-shikimate ester to caffeoyl-CoA is also catalyzed by HCT.

Caffeoyl CoA is next converted into feruloyl-CoA *via* methylation of the 3-hydroxyl by caffeic acid *O*-methyltransferase (CMOT). It is noted that this enzyme also contributes to the defense systems in plants in addition to its involvement in phenylpropanoid biosynthesis (Wang et al., 2018). The resulting feruloyl-CoA is furthermore transformed by cinnamoyl-CoA reductase (CCR) to afford coniferaldehyde. Ferulate 5-hydroxylase (F5H) then adds a hydroxyl group onto the coniferaldehyde at the 5-position to provide 5-hydroxyconiferaldehyde. The 5-hydroxyl is then methylated by CMOT to yield sinapaldehyde. Finally, sinapate is formed from sinapaldehyde in the presence of reduced epidermal fluorescence 1 aldehyde dehydrogenase (Nair et al., 2004).

**FIGURE 9:**
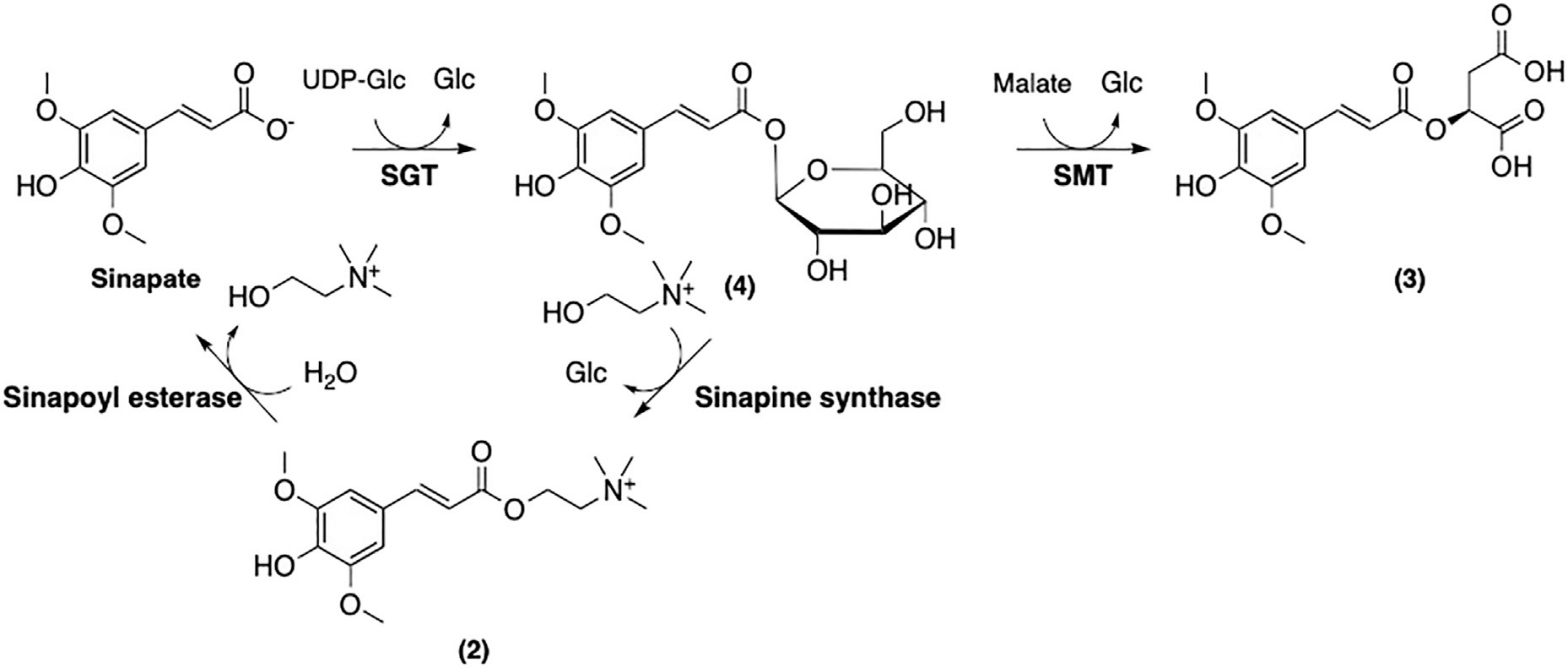
Biosynthetic modification of sinapate to afford three main SinE in plants.

Further modifications of sinapate yield three main sinapoyl esters including sinapine (**2**), sinapoyl malate (**3**), and sinapoyl glucose (**4**) (Figure 9). It has been suggested that sinapoylglucose:malate sinapoyltransferase (SMT) is also responsible for the conversion of sinapate to **4** (Lorenzen et al., 1996). 1-*O*-Sinapoylglucose:choline sinapoyltransferase (also known as sinapine synthase) converts **4** to **2** (Vogt et al., 1993). Sinapoycholine esterase can also convert **2** back to **1** in order to provide the required amount of choline during the seedling stage (Clauß et al., 2011). On the other hand, replacing the glucose moiety of **4** by malate is catalyzed by sinapoylglucose:malate sinapoyltransferase (SMT), producing sinapoyl malate (**3**) (Lorenzen et al., 1996). By using these three sinapoyl esters as the main building blocks, plants produce a broad range of SinEs that are involved in many different biological processes (Nićiforović and Abramovič, 2014).

## Chemical Synthetic Pathway of Sinapic Acids and Derivatives

### Sinapic Acid

**FIGURE 10:**
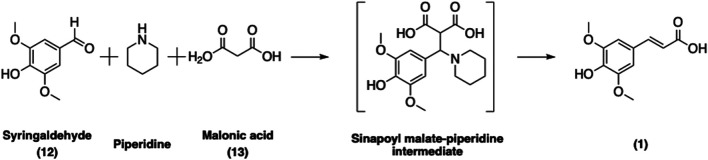
Synthesis of SinA *via* a sinapoyl malate-piperidine intermediate following the Knoevenagel-Doebner condensation approach.

SinA can be readily synthesized chemically *via* a Knoevenagel-Doebner condensation of syringaldehyde (**12**) and malonic acid (**13**) in piperidine (Figure 10) (Horbury et al., 2018; Flourat et al., 2020). Several greener approaches involving microwave activation (Mouterde and Allais, 2018) or L-proline as a catalyst in ethanol (Peyrot et al., 2019) have been developed in order to reduce the use of hazardous base and to enhance the overall yield and greenness of the synthetic process. Nevertheless, these improvements also have their own limitations. For example, substituting L-Pro for piperidine in ethanol requires an extra purification step by chromatography (Peyrot et al., 2019) whereas using piperidine as the catalyst requires only a simple acidic washing to afford pure SinA (Horbury et al., 2018). Taken together, the current protocols are straightforward and provide access to SinA; however further improvements should be made in order to enhance the greenness of the process.

### Sinapate Esters

#### Sinapoyl Choline or Sinapine

**FIGURE 11:**
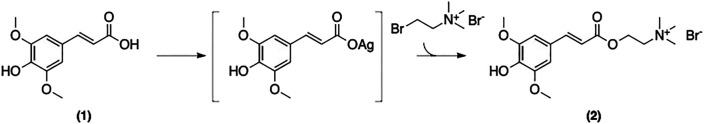
Synthesis of sinapine described by Clausen et al.

Sinapine is omnipresent in Brassica plants. The first synthetic approach to **2** was reported by Clausen et al. (Figure 11) (Clausen et al., 1982; Clausen et al., 1983). Using SinA isolated from *Sinapis Alba* L., and AgNO_3_, the corresponding SinA-Ag complex was reacted with bromocholine bromide to afford the pure product after chromatographic purification. Although pure sinapine was obtained, there were several drawbacks to this approach including low overall yield, toxic reagents and waste-generating purification steps.

**FIGURE 12:**
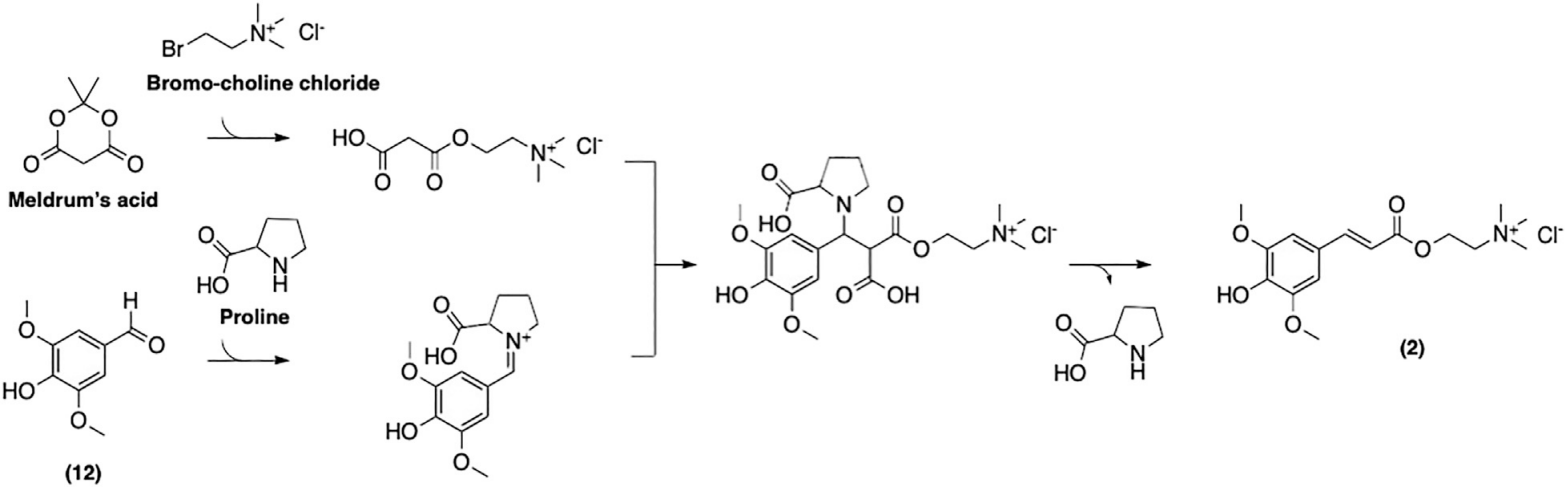
Synthesis of sinapine described by Mouterde et al.

Mouterde et al. have recently reported a more straightforward multigram-scale synthetic process for (**2**) (Figure 12) (Mouterde et al., 2020). Their approach relies on the well-established Knoevenagel-Doebner condensation of syringaldehyde and choline malonate. This two-step strategy gives access to desired SinE in a decent overall yield, while avoiding the use of toxic reagents. This enhances both the cost-efficiency and the environmental friendliness of the process. Moreover, this method was reported to be applicable to other naturally occurring *p*-hydroxycinnamic acids such as coumaric, caffeic, and feruloyl acids. We believe that this approach is, to date, the most cost- and time-efficient protocol as well as the most attractive in the context of green chemistry.

#### Sinapoyl Glucose

Sinapoyl glucose is the precursor of sinapoyl malate **(3)** in SinE biosynthesis (Figure 9). The enzymatic conversion of *p*-hydroxycinnamic acid into the corresponding glucose derivative using recombinant *Gomphrena globosa* sinapate glucosyltransferase was studied by Matsuba et al. (Matsuba et al., 2008). This biochemical approach was applicable to most naturally occurring *p*-hydroxycinnamic acids such as ferulic acid, caffeic acid, 4-coumaric acid, and SinA. Unfortunately, the reported yield was low for sinapoyl and feruloyl glucose.

**FIGURE 13:**
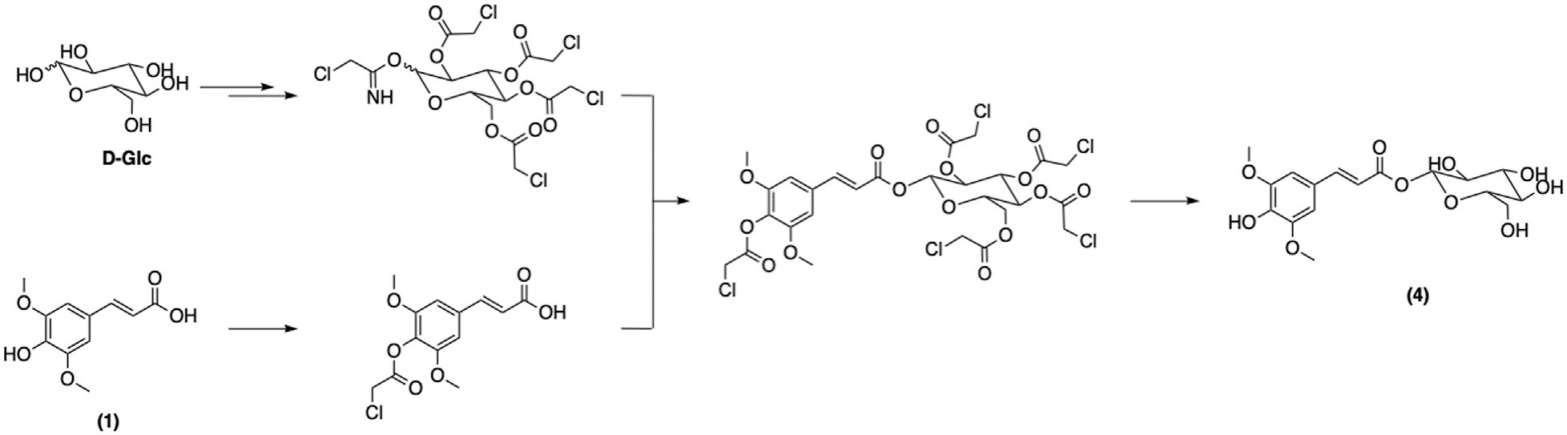
Synthesis of sinapoyl glucose described by Zhu et al.

Zhu et al. therefore devised another synthetic strategy to overcome the limitations of the previous method (Zhu and Ralph, 2011). The authors carried out a stereoselective glycosylation between a protected glycosyl donor and 4-*O*-chloroacetylated *p*-hydroxycinnamic acids (either ferulic or sinapic acid) (Figure 13). The subsequent cleavage of the chloroacetyl groups was then performed under mild conditions to yield desired sinapoyl or feruloyl glucose derivatives. This method successfully furnished the sinapoyl glucose (**4**) in high yields. To the best of our knowledge, this synthetic strategy remains the most efficient way to obtain **4**. Nevertheless, one drawback is that this synthesis requires multiple protection/deprotection steps for both the sugar and the *p*-hydroxycinnamic moieties.

#### Sinapoyl Malate

**FIGURE 14:**
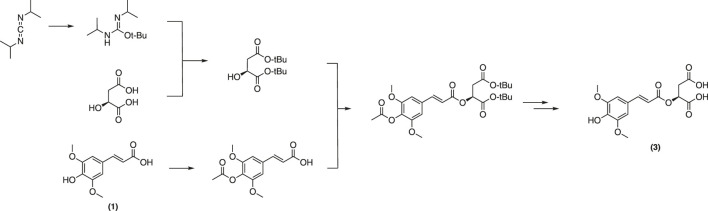
Synthesis of sinapoyl malate described by Allais et al.

Biosynthesized from sinapoyl glucose (**4**) *in planta*, sinapoyl malate (**3**) is crucial for regulating lignin biosynthetic enzymes in plants (Goujon et al., 2003). A total synthesis of **3** was reported by Allais et al. (Allais et al., 2009). This strategy employed a convergent approach from sinapic acid (**1**) and the corresponding protected malate moiety to afford the desired malate ester (Figure 14).

**FIGURE 15:**
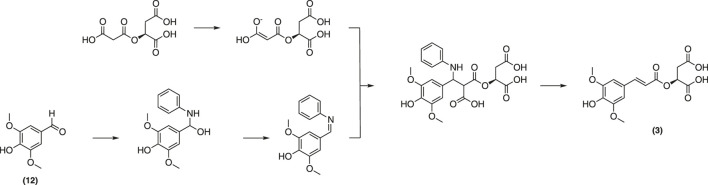
Synthesis of sinapoyl malate described by Peyrot et al.

Although pure final product was obtained with a decent yield, the extensive use of toxic solvents along with multiple protection/deprotection steps throughout the pathway will likely hinder of the application of this approach at multigram-scales. With this in mind, Peyrot et al. have devised a more sustainable and straightforward, protecting group-free procedure based on the Knoevenagel-Doebner condensation of syringaldehyde (**12**) and malic monomalonate ester (Figure 15) (Peyrot et al., 2020b). Sinapoyl malate and analogues were thereby obtained in higher yields. In addition, the method is more environmentally friendly as it avoids toxic solvents and reagents as well as waste-generating protection/deprotection steps. It is noteworthy to mention that sinapoyl malate (**3**) also helps plants to protect themselves from UV radiations.^58^

#### Other Sinapoyl Esters

**FIGURE 16:**
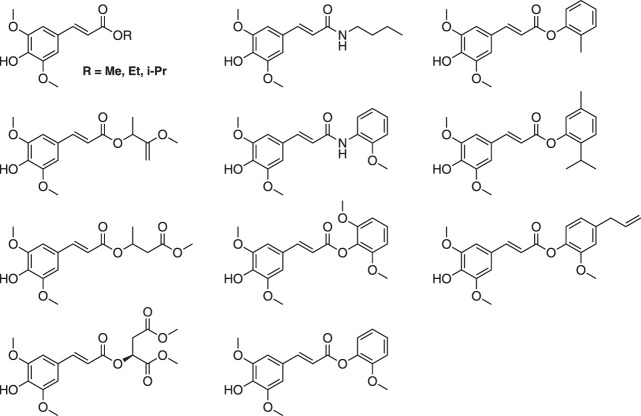
Several structural examples of other sinapate esters.

Other synthetic SinEs are of great interest with regard to their photophysical and biological properties (Dean et al., 2014; Baker et al., 2018; Peyrot et al., 2020a). Most SinEs are obtained *via* Knoevenagel-Doebner condensations (Baker et al., 2018; Peyrot et al., 2020a). The is more advantageous than direct acid-catalyzed esterification of SinA, as it enables access to a larger range of SinEs while remaining simple and ecologically attractive (e.g., no protection/deprotection sequences). Some structural examples are shown in Figure 16.

## Recovery of and Purification of Sinapic Acid and Derivatives From *Brassica* Biomass

### Recovery of Sinapic Acids and Derivatives From *Brassica* Biomass

The extraction of bioactive molecules from agro-industrial wastes has drawn increasing attention (Chuo et al., 2020; Flourat et al., 2020). The recovery of SinA and derivatives mainly relies on solid-liquid extraction where water/alcohol mixtures are often used as the extraction solvent (Prapakornwiriya and Diosady, 2014; Flourat et al., 2019; Laguna et al., 2019; Reungoat et al., 2020). Despite its popularity, only few optimization studies of the extraction process under these conditions have been reported. Indeed, most of the authors use high temperatures (close to the boiling point) to increase the yields of SinA and derivatives. However, Flourat et al. have optimized extraction conditions of sinapine from mustard bran using response surface methodology. An extraction temperature of 55°C with a concentration of 66% ethanol represents their optimal conditions (Flourat et al., 2019). Another optimization study revealed that 75°C and 55% ethanol lead to the highest yield from mustard bran (Reungoat et al., 2020). In summary, optimal conditions to recover SinA and SinEs from *Brassica* biomass must be considered as around 60% of alcohol and temperature ranged from 50°C to boiling point of using alcohol. Although water/methanol mixtures have been conventionally used to recover phenolics from processed biomass (Lin and Harnly, 2010; Prapakornwiriya and Diosady, 2014; Laguna et al., 2018), aqueous ethanol mixtures are more attractive thanks to the low toxicity of ethanol over the more hazardous methanol (Flourat et al., 2019; Reungoat et al., 2020).

Solid-liquid extraction using water/alcohol mixtures remain the conventional method to recover phenolic compounds from *Brassica* biomass thanks to its simplicity, time, and cost efficiency. This method, however, requires an additional purification step, as the use of mixture alcohol/water also extracts other non-phenolic compounds such as proteins, glucosinolates, carbohydrates and many other water-soluble chemicals. A more selective and straightforward recovery method of these secondary metabolites remains to be established.

An innovative recovery of SinA and innate SinE under corresponding alkyl ester form using different alcohols has been reported by Li and Guo (Li and Guo, 2017). Base-catalyzed alcohol extraction of rapeseed meal was conducted and followed by a purification by column chromatography with silica as stationary phase. The recovery of SinE (methyl sinapate) was reported to be up to 7.2 mg/g of rapeseed meal. Several alkyl SinEs (including ethyl, propyl, butyl, hexyl, octyl, decyl and dodecyl sinapate) were obtained through this method; however, their purification proved difficult due to the similar polarity of the alcohol and the corresponding alkyl sinapate ester. It is worth mentioning that the subsequent valorization of carbohydrates and residual meal was included into the extraction process. In summary, this method allows to simultaneously isolate desired phenolic compounds under corresponding ester forms and other valuable components from rapeseed meal.

### Intensified Recovery of Sinapic Acid and Derivatives Using Physical Accelerators

Intensified water/alcohol extraction techniques enhance the recovery of secondary metabolites from various *Brassica* biomass samples (Sparr Eskilsson and Björklund, 2000; Li et al., 2010; Dubie et al., 2013; Nandasiri et al., 2019). These advanced technologies are more time- and energy-efficient as compared to conventional extraction methods since they reduce the extraction temperature as well as the amount of extraction solvent required. This avoids the need for high alcohol concentrations and extended extraction durations. In this context, we provide in this section relevant examples of intensified SinA and derivative recovery from many that have reported in the literature.

Physical accelerators, such as ultrasound, have been employed in a number of studies (Dubie et al., 2013; Szydlowska-Czerniak and Tulodziecka, 2015; Yu et al., 2016). Dubie et al. reported that low-frequency, high-intensity ultrasound treatment (20 kHz and 0.5 W/ml) of *B. juncea* meal improves the aqueous ethanol extraction. Several parameters, i.e., extraction temperature, ethanol concentration, sonication duration, and solvent/material ratio were subjected to a one-factor-at-a-time optimizations (Dubie et al., 2013). The results show that the extraction of SinA and derivatives under mild conditions (70% EtOH/water for 30 min at 25°C) yields comparable results to the conventional water/ethanol extraction that require an extended extraction time (70% EtOH for 7 days at room temperature). These results furthermore confirm the interest of this intensification strategy.

Microwave-accelerated extraction also enhances the recovery of bioactive molecules by increasing the motion of free water molecules within the plant tissue which then releases the target metabolites (Sparr Eskilsson and Björklund, 2000). This extraction method is believed to be advantageous compared to the conventional method (Jokić et al., 2012; Yang et al., 2014; Zago et al., 2015; Yu et al., 2020). Jokic et al. studied the extraction of phenolics from broccoli using microwave treatment with aqueous methanol under optimal conditions (Jokić et al., 2012). Microwave irradiation reduces extraction time while enhancing the phenolic concentration in the extracts. Unfortunately, the relatively high cost of the microwave apparatus, along with undesired chemical reactions due to the application of high temperatures during the extraction hinders wider use of this method, despite its benefits (Khoddami et al., 2013).

Accelerated solvent extraction (ASE) is a technique carried out under high pressure and an inert atmosphere with a range of extraction temperatures from 35 to 200°C. This intensification method has been applied to recover secondary metabolites from *Brassica* biomass (Mohn et al., 2007; Blažević et al., 2020; Nguyen et al., 2020), including SinA and its derivatives (Li and Guo, 2016; Nandasiri et al., 2019). Aqueous alcoholic extraction of phenolics at high temperature (140–180°C) and high pressure resulted in better extraction yield than conventional method (Nandasiri et al., 2019). On the other hand, it was also reported that, under similar extraction conditions (200°C, 20 min), SinA was degraded into canolol through decarboxylation (Li and Guo, 2016). The relatively high cost of the extractor apparatus must also be considered a drawback for industrial scale applications.

Nowadays, supercritical carbon dioxide (Sc-CO_2_) as extraction solvent has become attractive as an environmentally friendly technique for the extraction of secondary metabolites. The advantages of using Sc-CO_2_ for phenolic extraction from canola press cake over conventional methods was reported by Li et al. (Li et al., 2010). The results show that Sc-CO_2_ extraction using ethanol as co-solvent enhances the extraction of phenolics, with ca. 10 mg of phenolics extracted per gram of dry matter from selected biomass. In addition, this extraction method appears to avoid the conversion of sinapine into SinA during the extraction process. The major drawback is the need for very specific technical expertise as well as material costs.

Advanced extraction techniques exhibit many advantages in terms of time, solvent consumption, and energy efficiency. Ultrasound-accelerated extraction, microwave-assisted extraction, and Sc-CO_2_ become more attractive in industrial scale whereas ASE is the most rapid and efficient method for recovering valuable chemicals from biomass in laboratory scale. Taken together, these intensification techniques allow a more profitable recovery of desired SinA and derivatives from selected *Brassica* biomass.

### Enzyme Assisted Recovery of Sinapic Acid and Derivatives

Carbohydrase (Viscozyme L.) and pectinase (Rapidase) were used to assist the recovery of phenolics from cauliflower (*B. oleracea* L var. *botrytis*) outer leaves by disrupting linkages between phenolics and cell-wall polymers (Huynh et al., 2014). In this study, cauliflower leaves were pretreated with either carbohydrase or pectinase prior to aqueous alcohol extraction of phenolics. Multiple extraction parameters related to the enzyme pretreatment step including type of enzyme, concentration, incubation temperature, pH, and time were studied. As a result, enhanced recovery yields were observed in enzyme pretreated samples.

Another study employing enzyme-assisted extraction of rapeseed meal was also disclosed by Laguna et al. (Laguna et al., 2019). Recombinant cinnamoyl or feruloyl esterase from *Aspergillus niger* was applied to the methanolic extract of rapeseed meal in order to hydrolyze ester linkages between *p*-hydroxycinnamic acids and carbohydrates. This enhanced the specific recovery of SinA.

Enzyme-assisted recovery becomes an attractive and environmentally friendly method to recover SinA and its derivatives from biomass. This approach is straightforward and accessible thanks to the convenient operating conditions. Nevertheless, the substrate specificity and the high cost of using enzymes limit the wide use of this methodology.

### Purification of Recovered Sinapic Acid and Derivatives

The use of mixture alcohol/water enables the extraction of other non-phenolic compounds such as proteins, glucosinolates, carbohydrates and many other water-, alcohol- and water/alcohol mixture soluble chemicals. Additional process is therefore necessary in order to recover SinA and derivatives at the necessary levels of purity.

For this, three technologies are commonly used: membrane processes, liquid/liquid extraction and adsorption chromatography. The latter is often employed with many studies reported in literature (Prapakornwiriya and Diosady, 2014; Thiel et al., 2015; Odinot et al., 2017; Laguna et al., 2018). Crude biomass extracts were adjusted to acid pH values prior to loading onto preparative ion exchange columns. Bound SinA was then eluted with an aqueous alcohol solution. Moreno-Gonzalez et al. have improved the binding capacity by studying a large range of anionic resins (Moreno-González et al., 2020). The authors further showed that, compared to the batch adsorption method, the column adsorption approach afforded higher selectivity towards SinA, which led to a higher recovery rate. Although the adsorption/desorption approach allows an efficient recovery of SinA on preparative scales, sinapine was not recovered in its native ester form, as this method takes advantage of charge differences between sinapine and SinA.

Liquid-liquid extraction (LLE) is commonly used as a preparation step, at the analytical stage, to measure the phenolic content in plant extracts. First step consists of increasing the partition coefficient by acidifying the extracts to pH 2 (Berthod and Carda-Broch, 2004). Aprotic organic solvents such as diethyl ether (DE) and ethyl acetate (EA) were then used to recover desired phenolics (Dabrowski and Sosulski, 1984; Galanakis et al., 2013). Unfortunately, this technique is solvent-consuming, and, therefore, does not fit into a sustainable context.

Membrane processes are also employed to isolate desired phenolic compounds from *Brassica* biomass (Xu and Diosady, 2002; Sinichi et al., 2019). Biomass was extracted under usual alkaline conditions and the extracts were then filtered multiple times through selected membranes until reaching the optimized purity. Adjusting pH to acid between filtration steps was often required to separate desired SinA from proteins and other undesired compounds (Xu and Diosady, 2002; Sinichi et al., 2019). The efficiency of these process was reported between 70 and 90%.

Although membrane processes are rapid and efficient purification techniques, however the purity is lower than that of adsorption chromatography. Hence, the adsorption chromatography, despite being cost- and time consuming, remains the conventional purification method for SinA and derivatives.

### Biological Activities

Along with other ubiquitous *p*-hydroxycinnamic acids in the plant kingdom, SinA and its derivatives have been extensively studied regarding their biological activities (Neelam et al., 2020; Sova and Saso, 2020). Mainly recognized as potent antioxidant reagents, these metabolites are particularly of interest regarding their antibacterial and UV-filter properties along with many other health benefits (Taofiq et al., 2017; Sova and Saso, 2020). Here, biological activities and properties of SinA and its main corresponding SinEs including sinapine, sinapoyl malate, and sinapoyl glucose, are discussed and summarized in Table 1.

**TABLE 1:** Biological activities and properties of SinA and SinEs reported in this review.

Metabolite	Biological activities or properties	Effects	Reference(s)
SinA (**1**)	Antioxidant	Good ABTS scavenging activity	Hussain et al. (2020)
	Antioxidant	DPPH, ABTS, hydroxyl, and superoxide radical scavenging	Mathew et al. (2015)
	UV-filter	Good absorption activity within UV-B region	Dean et al. (2014)
	Antibacterial	Inhibition on polygalacturonase- (54%) and polygalacturonic acid lyase activities (43%) from *Erwinia cartovora* subsp. *carotovra* at 400 μg/ml	Lyon and McGill (1989)
	Antibacterial	High antibacterial activity of extract from rapeseed flour against different strains of *Escherichia coli*	Nowak et al. (1992)
	Anti-inflammatory	Inhibition on different proinflammatory factors such as nitric oxide synthase, cyclooxygase 2, and proinflammatory cytokines via Factor-ΚB inactivation	Yun et al. (2008)
	Anti-inflammatory	Inhibition of monocyte adhesion to lipopolysaccharide-stimulated endothelial cells	Calabriso et al. (2019)
	Anticancer	Cytotoxicity and anti-angiogenic activity of SinA-copper oxide nanoparticles	Raj Preeth et al. (2019)
	Anticancer	Antitumor activity against colon (Caco-2) and cervical (HeLa, SiHa, and C33a) human cancer cell lines of extract from *Butia odorata* noblick fruit	Boeing et al. (2020)
	Antidiabetic	Amelioration of hyperglycemia in streptozotocin-induced type 1-like diabetic rats	Cherng et al. (2013)
	Antidiabetic	Prevention of the progression of diabetes mellitus in streptozotocin-induced type 2 diabetic rats	Alaofi (2020)
	Antihypertensive	Effects on systolic blood pressure by attenuating fibrosis and oxidative stress	Silambarasan et al. (2014)
	Anti-anxiety	Anxiolytic property mediated via GABA_A_ receptor in mice	Yoon et al. (2007)
Methyl/Ethyl sinapate (structure shown in Figure 16)	UV-filter	UV-photostability and absorption of *cis*-and *trans-* isomers	Baker et al. (2018), Horbury et al. (2018)
Sinapine (**2**)	Antioxidant	Good ABTS scavenging activity	Hussain et al. (2020)
	Antioxidant	33.2 and 88.4% at a molar ratio of SinA to DPPH^•^ of 0.2 and 0.5, respectively	Kikuzaki et al. (2002), Nenadis and Tsimidou (2002)
	Antimicrobial	Excellent antimicrobial activity against *Escherichia coli* K12 strain at 10%w concentration	Mouterde et al. (2020)
Sinapoyl malate (**3**)	Antioxidant	DPPH scavenging (EC_50_ = 10.6 nmol)	Peyrot et al. (2020b)
	UV-filter	Good UV-absorption activity within UV-A (315–400 nm) and UV-B (280–315 nm) regions	Peyrot et al. (2020a), Peyrot et al. (2020b)
	UV-filter	Good absorption activity within UV-B region	Dean et al. (2014)
	Antibacterial	Comparable antibacterial activity to phenoxyethanol	Peyrot et al. (2020b)
Sinapoyl glucose (**4**)	Antioxidant	35.8 mM to scavenge 25 × 10^18^ DPPH radicals	Thiyam et al. (2006)

### Antioxidant Activity

Free radical and other oxidizing reagents are generated during metabolic processes. These compounds lead to oxidative stress in the body and are often associated to numerous human diseases (Taofiq et al., 2017; Sova and Saso, 2020). Antioxidant reagents scavenge these free radical and oxidizing reagents, thus efficiently reducing their harmful effects (Cartea et al., 2011).

*p*-Hydroxycinnamic acids including SinA and corresponding SinEs are prominent as potent antioxidants. The radical scavenging activity of SinA for 2,2-diphenyl-1-picrylhydrazyl (DPPH^•^) was determined to be 33.2 and 88.4% at a molar ratio of SinA to DPPH^•^ of 0.2 and 0.5, respectively (Kikuzaki et al., 2002; Nenadis and Tsimidou, 2002). Sinapine, on the other hand, exhibits even higher antioxidant activity than SinA (Thiyam et al., 2006). The antioxidant activities of sinapoyl malate and sinapoyl glucose were reported to be comparable to conventional antioxidants such as butylated hydroxyanisol (BHA), butylated hydroxytoluene (BHT), or trolox (Thiyam et al., 2006; Peyrot et al., 2020b). In addition, antioxidant activity of Canola meal extract was also studied by Hussain et al. (Hussain et al., 2020). Their results showed that SinA and sinapine exhibited good radical scavenging activity towards 2,2′-azinobis (3-ethylbenzothiazoline-6-sulfonic acid) (ABTS). We highly recommend reviews by Niciforovič and Abramovič (Nićiforović and Abramovič, 2014) and Chen (Chen, 2016) for further details concerning the free radical scavenging activity of SinA and other derivatives toward other free radical molecules such as superoxide anion radicals, hydroperoxyl radical, hypochlorite, and nitric oxide.

The antioxidant property of *p*-hydroxycinnamic acids mainly relies on the hydroxyl group at the *para-*position (Pei et al., 2016). Besides this characteristic functional group, addition of extra hydroxyl groups on to the phenyl core allows higher radical and oxidizing reagent scavenging activities. For instance, caffeic acid shows better antioxidant activity than that of SinA (Mathew et al., 2015). Furthermore, extra methoxyl group on the *p*-hydroxycinnamic acid core further improves the radical scavenging activities of these metabolites (Mouterde et al., 2020).

### UV-Filter Activities

Sunscreen lotions are often advised to avoid permanent skin damages due to long-term light exposure. *p*-hydroxycinnamic acids such as ferulic and caffeic acid are supplemented in these cosmetical products to improve skin protection efficiency (Kumar and Pruthi, 2014; Taofiq et al., 2017; Coman and Vodnar, 2020).

As prominent *p*-hydroxycinnamic acid, SinA and its derivatives also exhibit high photo-stability and UV-absorption (Dean et al., 2014; Horbury et al., 2019; Peyrot et al., 2020a). The photophysical properties of numerous synthetic SinEs were recently studied by Peyrot et al. (Peyrot et al., 2020a; Peyrot et al., 2020b) who have reported comparable, or even better, photo-activities of these synthetic *p*-hydroxycinnamic acids than octinoxate (a conventional fossil-based UV-filter reagent) in term of UV absorption and photostability.

It is noteworthy that the structural conformation of these metabolite plays an important role in the UV activities. The activities of SinA and its derivatives are mainly attributed to the *trans*-isomers, whereas the *cis*-isomers have shown limited absorption thresholds (Baker et al., 2018; Horbury et al., 2018) and exhibit genotoxicity activity (Sharma et al., 2017). In order to address this symmetric drawback, addition of an acrylic functional group by the esterification of SinE allows to negating the aforementioned negative effects (Horbury et al., 2019).

### Antimicrobial Activity

The antimicrobial activities of SinA and its derivatives have also been well studied. In an early study (Lyon and McGill, 1989), the antimicrobial activity of SinA against *Erwinia carotovora* subsp. *carotovora* which causes foodborne illness in root vegetables was reported. Inhibition of a broad range of Gram-negative and Gram-positive bacteria have also been demonstrated using a SinA fraction isolated from the ethanolic extract of rapeseed (Nowak et al., 1992). The antimicrobial activities of sinapine (Mouterde et al., 2020) and other SinEs (Peyrot et al., 2020b) against *Escherichia coli* have also been recently highlighted. These literature reports strongly suggest that SinA and its derivatives are potential biobased antimicrobial reagents.

### Other Health Benefits

Many human health benefits of SinA and derivatives have been reported, and these include anti-inflammatory (Yun et al., 2008; Calabriso et al., 2019), anticancer (Raj Preeth et al., 2019; Boeing et al., 2020), anti-diabetic (Cherng et al., 2013; Alaofi, 2020), and antihypertensive properties (Silambarasan et al., 2014) as well as their protections of the nervous, respiratory, and digestive systems (Sova and Saso, 2020). For further details on the health benefits of these metabolites, we highly recommend the reviews by Sova and Saso (Sova and Saso, 2020) and by Neelam et al. (Neelam et al., 2020).

## Conclusion

The therapeutic and biological benefits of SinA and its derivatives have been extensively studied. Although the use of advanced extraction techniques to recover these metabolites remains limited, mainly due to their relatively high cost, the accessibility of these metabolites from biomass extraction has been improved. Meanwhile, chemical synthesis of natural and non-natural SinEs through sustainable approaches have been devised to provide a straightforward access to these molecules while taking into account the environmental impacts of the processes. Biochemical studies of SinA and its derivatives have been also been extended to provide crucial information concerning their innate accumulation and their important biological roles in plants.

As mentioned above, SinA and its common derivatives including sinapine, sinapoyl malate, and sinapoyl glucose, exhibit many valuable properties for human health beyond their well-known antioxidant and antibacterial activities. Their photo-physical properties are also important for applications as biobased UV-filters. Further extended SinEs, with regards to their interesting biological activities, also represent attractive ingredients in the pharmaceutical, cosmetic and food industries.

We believe that SinA and derivates are prospective bio-based substitutes for conventional antioxidants with regards to their high antioxidant and antimicrobial activities, along with many other health benefits. These metabolites are furthermore potential sustainable and non-toxic alternatives to the conventional UV-filters that are currently flagged as human- and eco-toxic (Burnett and Wang, 2011; Krause et al., 2012). Despite several aforementioned limitations, SinA and its derivatives represent potential multifunctional chemicals with a bright future that deserves to be further investigated and developed.
